# Graphitic carbon grown on fluorides by molecular beam epitaxy

**DOI:** 10.1186/1556-276X-8-11

**Published:** 2013-01-03

**Authors:** Sahng-Kyoon Jerng, Jae Hong Lee, Yong Seung Kim, Seung-Hyun Chun

**Affiliations:** 1 Department of Physics and Graphene, Research Institute Sejong University, Seoul, 143-747, South Korea

**Keywords:** Graphene, Graphitic carbon, Molecular beam epitaxy, Fluoride, 81.05.uf, 81.15.Hi, 78.30.Ly

## Abstract

We study the growth mechanism of carbon molecules supplied by molecular beam epitaxy on fluoride substrates (MgF_2_, CaF_2_, and BaF_2_). All the carbon layers form graphitic carbon with different crystallinities depending on the cation. Especially, the growth on MgF_2_ results in the formation of nanocrystalline graphite (NCG). Such dependence on the cation is a new observation and calls for further systematic studies with other series of substrates. At the same growth temperature, the NCG on MgF_2_ has larger clusters than those on oxides. This is contrary to the general expectation because the bond strength of the carbon-fluorine bond is larger than that of the carbon-oxygen bond. Our results show that the growth of graphitic carbon does not simply depend on the chemical bonding between the carbon and the anion in the substrate.

## Background

From the success of graphene growth on Ni or Cu by chemical vapor deposition (CVD) [1,2], some variations were introduced to CVD to avoid the use of metallic catalysts [3-8]. However, the growth of carbon by chemical methods involves a complex mechanism due to the presence of carrier gases. For example, hydrogen acts as an etching reagent as well as a co-catalyst [9]. In contrast, physical deposition methods such as molecular beam epitaxy (MBE) are useful to understand the growth mechanism of carbon because of the relatively simple kinetics [10-13]. Experimentally, it has been shown that nanocrystalline graphite (NCG) could be formed on crystalline and amorphous oxides by direct sublimation of carbon [14-16]. Although first-principles calculations partly explained that the strong bonding between carbon and oxygen limited the cluster size [14,16], the growth mechanism is yet to be understood.

So far, carbon MBE has been tried on substrates containing elements from group IV [10-13], group V [17], and group VI [12,14-16]. Here, we present the results of carbon MBE on fluorides (where the anion belongs to group VII) and compare them with similar studies on oxides to understand the effect of the anion on the quality of NCG. Since the bonding between carbon and fluorine is much stronger than the bonding between carbon and oxygen, we expected the carbon film to be more amorphous. On the contrary, NCG of good crystallinity was formed on MgF_2_, and the cluster size deduced from Raman spectra was even larger than those of NCGs on MgO and sapphire [18,19]. These results show that the quality of NCG does not simply depend on the bond strength of carbon and substrate anion, and imply that the carbon growth mechanism could be more complex than previously thought.

## Methods

### Materials and film fabrication

Carbon MBE was done using a home-made ultra-high-vacuum MBE system and a carbon sublimation cell with a pyrolytic graphite filament. The pressure of the chamber was kept below 1.0×10^−7^ Torr during the growth by flowing liquid nitrogen in the shroud. Details about the growth procedure can be found elsewhere [14]. Fluoride substrates (MgF_2_(100), CaF_2_(100), and BaF_2_(111)) were purchased from a commercial vendor (CrysTec GmbH, Berlin, Germany). The growth temperature was fixed at 900°C because of the lower melting points of fluoride substrates compared to oxides.

### Characterization

Raman scattering measurements and spatial mapping were performed using a micro-Raman spectroscope (inVia system, Renishaw, Wotton-under-Edge, UK) operated by a 514.5-nm laser. A minimal laser power of 2 mW was used during the measurements to avoid any damage or heating of the carbon films. Atomic force microscopy (AFM) images were taken by a commercial system (NanoFocus Inc., Seoul, South Korea) in a non-contact mode. AFM in a contact mode was also used to determine the film thickness by measuring the step height after lithography. X-ray photoelectron spectroscopy (XPS) measurements to analyze carbon bonding characteristics were done using a Kratos X-ray photoelectron spectrometer (Kratos Analytical Ltd, Manchester, UK) with Mg Kα X-ray source. C1*s* spectra were acquired at 150-W X-ray power with a pass energy of 20 eV and a resolution step of 0.1 eV.

## Results and discussion

**Figure 1:**
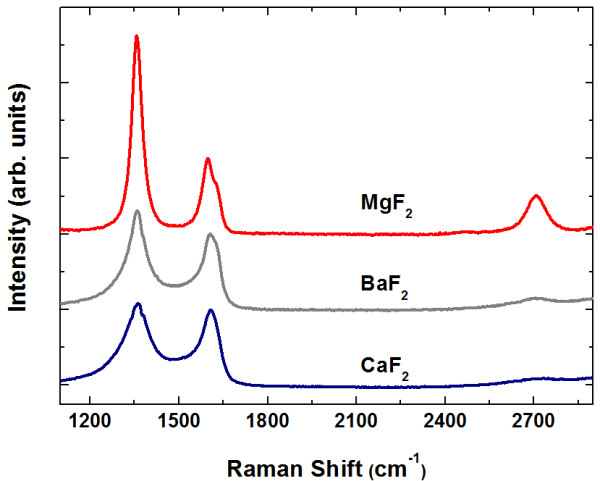
**Raman spectra of carbon films.** The films were grown by carbon MBE at 900°C on MgF_2_(100), CaF_2_(100), and BaF_2_(111). The pronounced 2D peak at approximately 2,700 cm^−1^ confirms that nanocrystalline graphite is formed on MgF_2_.

Figure 1 shows the Raman spectra from 3- to approximately 5-nm-thick carbon films grown on various fluorides by MBE. The characteristic peaks of graphitic carbon are well identified in all films: the D peak at approximately 1,350 cm^−1^ and the G peak at approximately 1,590 cm^−1^. These and previous studies show that MBE is an effective method for graphitic carbon growth on a wide range of substrates [14-17]. The degree of graphitization is, however, quite different depending on the cation. In fact, graphitic carbon refers to a wide range of disordered graphite, from NCG to mainly *sp*^2^ amorphous carbon. As clarified by Ferrari [20], the relative strength of D and G peaks alone cannot determine the degree of disorder, and it is the 2D peak at approximately 2,700 cm^−1^ which distinguishes NCG from amorphous carbon. As shown in Figure 1, the Raman spectra of the carbon film on MgF_2_ show a clear 2D peak, indicating that successful NCG growth was accomplished on MgF_2_ by carbon MBE. In contrast, the carbon films grown on CaF_2_ and BaF_2_ can be ascribed to amorphous carbon. As far as we know, carbon MBE on a family of substrates having the same anion has not been reported. Clear understanding of this cation dependence is yet to come, but our results will stimulate systematic studies on other series of substrates and further theoretical investigations.

**Figure 2:**
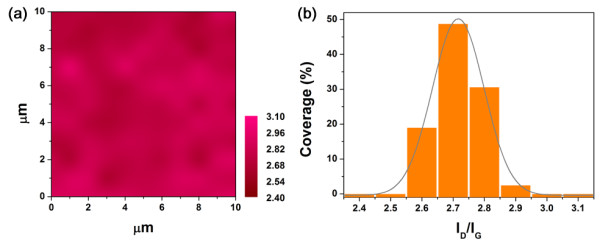
**Raman map of graphitic carbon on MgF**_**2**_**. (a)** The intensity ratio of the D peak to the G peak is mapped over 10 × 10 μm. The distributions, shown in **(b)**, imply a high spatial uniformity.

We will focus on the growth on MgF_2_ from now on and compare the results with NCGs on oxides. For a quantitative comparison, the Raman spectra of NCG on MgF_2_ were fit by several Lorentzian functions as in [15] (Table 1). Interestingly, the intensity ratios of the D peak and 2D peak to the G peak (*I*_D_/*I*_G_ and *I*_2D_/*I*_G_) are larger than those from NCG on MgO. Furthermore, all the peaks are narrower, implying a better crystallinity on MgF_2_ (from the comparisons of the full width at half maximum (FWHM) in Table 1 and those in [15]). The average cluster size, *L*_a_, can be calculated from the relation *I*_D_/*I*_G_ = *C L*_a_^2^, where *C* = 0.0055 and *L*_a_ in Å [20]. From *I*_D_/*I*_G_ = 2.7 (Table 1), we get *L*_a_ = 22 Å, a slight increase from those on oxides [15,16]. Figure 2 shows a Raman map of the intensity ratio of *I*_D_/*I*_G_ over 10 μm^2^. Most regions have *I*_D_/*I*_G_ = 2.7 ± 0.1, thus showing a high degree of uniformity. The uniformity is also better than that of NCG on MgO [16].

**Table 1:** **Fitting results of the Raman spectra from the graphitic carbon on MgF**_**2**_

	**D**	**G**	**2D**
Position (cm^−1^)	1,348	1,601	2,685
FWHM (cm^−1^)	44	61	83
*I*/*I*_G_	2.8	1	0.5

Lorentzian functions are used to fit D, G, and 2D peaks. FWHM, full width at half maximum.

All these results indicate that NCG on MgF_2_ is less disordered than those on oxides. This is quite surprising if we consider the bond strength of the C-F bond, which is larger than the C-O bond strength [18,19]. The high electronegativity of fluorine even makes the C-F bond partially ionic. From first-principles calculations, we have known that the strong C-O bond limits the cluster size of NCG on sapphire and MgO [14,16]. If that is the whole story, the stronger C-F bond should lead to smaller clusters on MgF_2_. Our results against this imply that an important factor is missing in the theoretical understanding of the NCG growth mechanism. Recently, models such as the catalytic role of step edges or the migration of cyclic carbons are good examples of pertinent suggestions [4,21].

**Figure 3:**
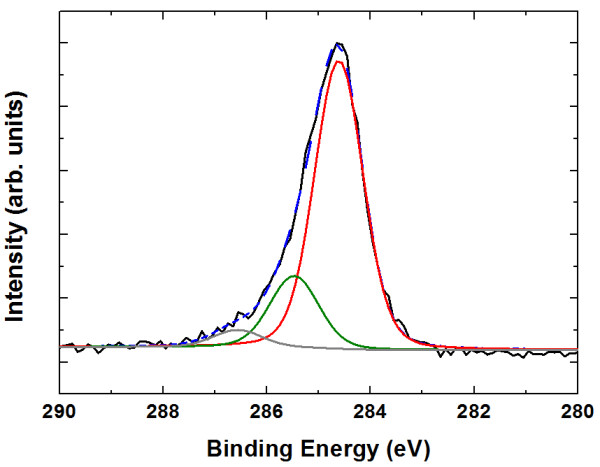
**C1*s*XPS spectra of graphitic carbon on MgF**_**2**_**.** The dashed line is a fit with four Lorentzian functions. The two strongest peaks (centered at 284.6 eV (red) and 285.8 eV (green)) are assigned to *sp*^2^ and *sp*^3^ hybridized carbon atoms, respectively. The fraction of the *sp*^2^ bond is estimated to be 80.1%.

Figure 3 presents XPS results to clarify the carbon bonding characteristics. Similar to previous studies [14,16], 284.7 ± 0.2 and 285.6 ± 0.2 eV components in C1*s* spectra are attributed to *sp*^2^ and *sp*^3^ bonds [22], namely, *sp*^2^ hybridization of carbon atoms and *sp*^3^ hybridization of C-C or C-H bonds, respectively [23]. The fitting results show that the fraction of the *sp*^2^ bond is more than 80%, confirming the NCG formation on MgF_2_.

**Figure 4:**
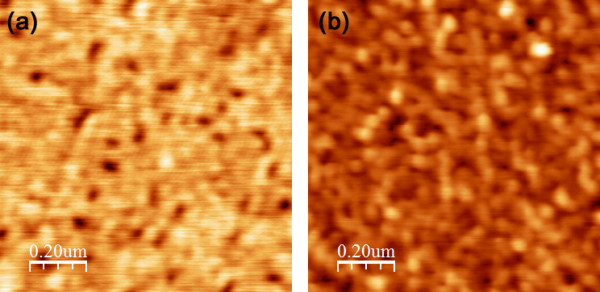
**AFM images of graphitic carbon on MgF**_**2**_**.** AFM images of 1 × 1 μm **(a)** before and **(b)** after the graphitic carbon growth on MgF_2_. The mean roughness parameters, *R*_a_, from 1 × 1 μm scans are **(a)** 0.97 and **(b)** 2.45 nm, respectively.

Finally, Figure 4 shows AFM images before and after the NCG growth on MgF_2_. Unlike crystalline and amorphous oxide substrates, the mean roughness parameter, *R*_a_, of the MgF_2_ substrate is large. The *R*_a_ of NCG (2.45 nm over 1 × 1 μm scan) is even larger by an order of magnitude than those NCGs on oxide substrates [14-16]. It is not clear why the surface morphology is worse while the Raman spectra indicate a better crystallinity. We hope that the understanding of NCG growth on MgF_2_ can lead to better NCG or possibly graphene growth on other (flat) dielectrics.

## Conclusions

In summary, we have grown graphitic carbon on fluoride substrates, expanding the application of carbon MBE into group VII anions. While amorphous carbons were formed on CaF_2_ and BaF_2_, nanocrystalline graphite of good crystallinity was formed on MgF_2_ despite the strong bonding between carbon and fluorine. In comparison to similar studies on MgO, the effect of the substrate anion on the quality of NCG contradicts the expectation based on the bond strength between carbon and the anion. Further systematic studies and theoretical investigations are encouraged to understand the carbon growth mechanism by MBE.

## Abbreviations

AFM: Atomic force microscopy; CVD: Chemical vapor deposition; FWHM: The full width at half maximum; MBE: Molecular beam epitaxy; NCG: Nanocrystalline graphite; XPS: X-ray photoelectron spectroscopy.

## Competing interests

The authors declare that they have no competing interests.

## Authors’ contributions

SKJ carried out the carbon molecular beam epitaxy experiments and X-ray photoelectron spectroscopy. JHL carried out the atomic force microscopy measurements. YSK characterized the thin films by Raman spectroscopy. SHC designed the experiments and wrote the manuscript. All authors read and approved the final manuscript.
